# Plant-derived natural products targeting inflammation in treatment of atherosclerosis

**DOI:** 10.3389/fphar.2025.1642183

**Published:** 2025-10-02

**Authors:** Zhen Hua, Xiaojie Wang, Le-Le Qin, Kun-Peng Zhu, De-Ye Li, Xin-Yue Zhang, Lei Zhang, Feng-Ting Zhai

**Affiliations:** ^1^ Department of Cardiology, Affiliated Hospital of Shandong University of Traditional Chinese Medicine, Jinan, China; ^2^ College of Traditional Chinese medicine, Shandong University of Traditional Chinese Medicine, Jinan, China; ^3^ First School of Clinical Medicine, Shandong University of Traditional Chinese Medicine, Jinan, China; ^4^ Department of Gynaecology, Affiliated Hospital of Shandong University of Traditional Chinese Medicine, Jinan, China

**Keywords:** plant-derived natural products, inflammation, atherosclerosis, traditional Chinese medicine, Chinese herbal medicine

## Abstract

Atherosclerosis (AS) is a complex and chronic inflammatory vascular disease, and it's also the pathological basis of various diseases such as coronary heart disease. Owing to their advantages such as multiple targets and rich diversity, Plant-Derived natural products with abundant resources will become an important source of anti-AS drugs. Herein, we explored the intricate association between inflammation and AS, focusing on plant-derived natural products that specifically target inflammation for the treatment of AS. This review systematically analyzes 10 plant species, focusing on 23 high-potential metabolites including Tanshinone IIA, Berberine, and Ginsenoside Rb1 et al. We found that natural products with anti-AS properties are primarily derived from plants belonging to the families Araliaceae, Umbelliferae, Fabaceae, and Lamiaceae. The chemical structures of these natural products mainly consist of flavonoids, alkaloids, saponins, and polysaccharides, among others. This suggests that natural products with different structural types can exhibit anti-AS activities, with flavonoids, alkaloids, and saponins showing greater potential. Major anti-inflammatory pathways center on NF-κB/NLRP3 inflammasome inhibition (e.g., Tan IIA, Salidroside), ROS scavenging (e.g., Danshensu, Rosmarinic acid), and endothelial protection (e.g., Astragaloside, Puerarin), while highlighting NLRP3 inflammasome inhibition and gut-microbiota modulation as emerging therapeutic avenues. These will actively promote the clinical application and industrial production of Plant Derived natural products, thereby developing anti AS drugs with excellent efficacy and reduced adverse reactions. We hope that this comprehensive review will provide valuable insights into the potential and pharmacological mechanisms of current natural medicines, thereby offering support for future drug discoveries.

## 1 Introduction

In recent years, there has been a rising incidence of atherosclerosis (AS), a complex and chronic inflammatory vascular disease that primarily affects the intimal and inner layers of arteries. AS-related diseases impose a significant economic burden on both the nation and society (Desai et al., 2021), while also increasing the risk of mortality for affected individuals. Based on the Global Burden of Diseases 2021 dataset, the prevalence rates per 100,000 population in China for atherosclerotic disease in 2021 were quantified as 3,042.3 cases of ischemic heart disease, 1,301.4 cases of stroke, and 1,331.1 cases of lower extremity peripheral artery disease. Extensive research has indicated that inflammation plays a pivotal role in the initiation and progression of AS plaques (Ross, 1999; Mahdavi-Roshan et al., 2025; Yamaura et al., 2025). Animal studies confirm that inflammatory factors can be locally administered to the vascular intima or adventitia. Such administration initiates or accelerates AS development. Subsequent human studies have highlighted the presence of inflammatory phenomena throughout the course of AS and across its various phenotypes. Acute coronary syndrome, for instance, exhibits a strong inflammatory background, whereby inflammatory phenomena are activated and contribute to plaque rupture or erosion; A retrospective cohort study demonstrated that elevated systemic inflammatory response index levels are independently associated with an increased incidence of carotid atherosclerosis (Nai et al., 2025). Notably, histological and hematological investigations have revealed significant accumulation and elevation of inflammatory factors in the tissues and bloodstream of patients with stable coronary heart disease. Furthermore, other distinctive phenotypes of coronary artery disease, such as variant angina and coronary calcification, are also influenced by inflammatory mechanisms (Ross, 1999; Li J. et al., 2022; Avula et al., 2025).

Natural products encompass a wide range of substances derived from plants, animals, and minerals. Traditional Chinese medicine, commonly referred to as “Chinese herbal medicine,” has a rich historical background and predominantly utilizes plant-derived natural products. In recent years, numerous researchers have observed that certain plant-derived natural products, including those utilized in traditional Chinese medicine, have the potential to modulate inflammation, thereby mitigating the formation and progression of AS and improving disease outcomes. These natural products exhibit effects beyond single pathogenic mechanisms and possess multitarget properties, targeting multiple pathways to ameliorate AS (Meng et al., 2022; Wang et al., 2022). Consequently, this comprehensive review aims to explore the intricate association between inflammation and AS, focusing on plant-derived natural products that specifically target inflammation for the treatment of AS. The remainder of this review will discuss representative TCM-derived plants, grouped by chemical class. It is hoped that this review will provide novel insights and perspectives for the prevention and treatment of AS.

### 1.1 Salvia miltiorrhiza Bunge [Lamiaceae; Salviae miltiorrhizae radix et rhizoma]

This product comprises dried roots and rhizomes of *Salvia miltiorrhiza Bge.*, a member of the Lamiaceae family. According to traditional Chinese medicine, it possesses blood-activating and stasis-dispersing properties. Modern pharmacological research has demonstrated that *S. miltiorrhiza* and its metabolites exhibit functions such as coronary artery dilation, myocardial ischemia prevention, and improvement of microcirculation. These impressive effects make it highly applicable in the prevention and treatment of cardiovascular diseases, thus finding extensive clinical utilization. The chemical composition of *S. miltiorrhiza* can be segregated into two main classes: liposoluble and water-soluble metabolites. The liposoluble metabolites include tanshinone I, dihydrotanshinone I, tanshinone IIA, tanshinone IIB, miltirone, nortanshinone, miltirone II, and hydroxytanshinone. Watersoluble (phenolic acid) metabolites encompass Danshensu, salvianolic acid A, salvianolic acid B, lithospermic acid, protocatechuic aldehyde, and rosmarinic acid. Research has revealed that multiple metabolites exhibit notable anti-AS activities.

#### 1.1.1 Tanshinone IIA

Tanshinone IIA (Tan IIA) is one of the main liposoluble metabolites of *S. miltiorrhiza*, exhibiting excellent anti-AS effects. It primarily functions in the treatment of AS-related diseases through its antioxidant and anti-inflammatory activities. Xu et al. (2011) discovered that Tan IIA significantly reduces the size of AS lesions in the aortic sinus and aortic intima of ApoE^−/−^ mice fed a high-cholesterol diet, with a decrease in macrophage infiltration within the plaque area. *In vitro* studies also revealed that Tan IIA significantly inhibits reactive oxygen species production induced by oxidized low-density lipoprotein (oxLDL) and the expression and activity of pro-inflammatory cytokines including Interleukin (IL)-6, Tumor Necrosis Factor (TNF)-α, Monocyte Chemoattractant Protein-1 (MCP-1) and Matrix Metalloproteinase (MMP)-9. Consistent findings from *in vivo* experiments indicated that Tan IIA significantly reduces arterial lipid deposition and lowers serum levels of Total cholesterol, Triglyceride, low-density lipoprotein cholesterol, Very low-density lipoprotein (Liu et al., 2015). Ren et al. (2010) further confirms that Tan IIA reduces the expression of MCP-1, Transforming Growth Factor-β1 (TGF-β1), and macrophage infiltration, while also decreasing TNF-α expression and NF-κB activation, suggesting that the cardioprotective effects of Tan IIA may be attributed to its ability to inhibit inflammatory responses. Multiple studies have demonstrated that, during AS intervention, Tan IIA significantly reduces the expression of pro-inflammatory cytokines, as well as the activity of MMP-2 and MMP-9 (Xuan et al., 2017; Fang et al., 2008; Wang et al., 2017). Li HZ reported that Tan IIA inhibits the maturation of antigen-presenting cells (including monocytes and dendritic cells) and reduces the expression of pro-inflammatory cytokines, while also weakening their ability to stimulate T-cell proliferation and cytokine secretion (Li et al., 2014). Wen J et al. found that Tan IIA significantly inhibits NOD like receptor hot protein domain related protein 3 (NLRP3) inflammasome activation induced by oxLDL, thereby improving AS formation and showing an association with the inhibition of NF-κB activation (Wen et al., 2020). Furthermore, Wang J et al. reported that Tan IIA reduces blood lipid levels, stabilizes AS plaques, reduces endothelial and inflammatory damage, and exerts anti-AS effects through the activation of the TGF-β/PI3K/Akt/eNOS pathway (Wang J. et al., 2020). Chen W et al. discovered that Tan IIA intervention in AS involves signaling pathways such as Ras, Rap1, MAPK, cAMP, and T-cell receptor (Chen et al., 2020). Moreover, Chen W et al. found that Tan IIA can alleviate AS by inhibiting miR-375 activation and enhancing macrophage autophagy and M2 polarization through the activation of Kruppel-like factor 4 (Chen W. et al., 2019). Similarly, Zhang et al. demonstrated that tanshinone IIA attenuates atherosclerosis by suppressing pro-inflammatory cytokines and downregulating inflammation-associated markers (iNOS, VCAM-1), primarily via inhibition of the MAPKs/NF-κB signaling pathway. This mechanistic insight further suggests a potential therapeutic strategy for concurrent atherosclerosis and hepatic steatosis. It underscores the critical interplay between inflammatory responses and dyslipidemia (Zhang Y. et al., 2025; Chen et al., 2024).

#### 1.1.2 Danshensu

Danshensu (DSS) is a water-soluble active metabolite extracted from *S. miltiorrhiza* and is widely used in the treatment of cardiovascular diseases in China. Through *in vivo* and *in vitro* experiments, Song Q et al. discovered that DSS prevents the progression of AS lesions induced by a high-fat diet in rats (Song et al., 2019). It reduces lipid deposition in the rat aorta, as well as serum levels of triglycerides, total cholesterol, low-density lipoprotein, and high-density lipoprotein in AS rats. Moreover, it decreases the levels of pro-inflammatory cytokines, including IL-1β, IL-6, TNF-α, Intercellular Cell Adhesion Molecule-1 (ICAM-1), and Vascular Cell Adhesion Molecule-1 (VCAM-1), and this effect may be mediated through inhibition of the TLR4/NF-κB pathway, highlighting the protective role of DSS in AS and its potential mechanism linked to inflammation inhibition. Additionally, Ye T et al. reported that water extract of *S. miltiorrhiza* significantly inhibits the expression of pro-inflammatory cytokines, reducing levels of pro-inflammatory cytokines. Through identification, it was confirmed that DSS possesses a bioactive metabolite with anti-inflammatory effects, possibly through the inhibition of downstream MAPK signaling pathways under the TNF cascade, where IL-6 acts as a key effective molecule and phosphorylation of ERK and JNK is suppressed (Ye et al., 2020).

#### 1.1.3 Rosmarinic acid

Rosmarinic acid is a polyphenol present in *S. miltiorrhiza*, which exhibits antioxidative, anti-inflammatory, and insulin-sensitizing properties. A study conducted by Nyandwi JB et al. revealed that rosmarinic acid exerts preventive effects against AS by reducing the secretion of IL-1β through the downregulation of the p38-FOXO1-TXNIP pathway and the inhibition of NLRP3 inflammasome activation (Nyandwi et al., 2020).

#### 1.1.4 Sodium danshensu

Sodium danshensu, a phenolic aromatic acid metabolite primarily derived from the roots of *S. miltiorrhiza*, is commonly employed in the treatment of cardiovascular disorders, including angina pectoris. Its documented pharmacological activities encompass the inhibition of platelet aggregation, improvement of microcirculation, regulation of lipid metabolism, and attenuation of atherosclerosis. Sodium danshensu exerts protective effects against atherosclerosis by significantly reducing aortic atherosclerotic plaque area, lowering lipid levels, and attenuating plaque progression. The underlying mechanism involves the suppression of macrophage inflammation via modulation of the NF-κB signaling pathway (Zhang X. et al., 2025; Zeng et al., 2023).

### 1.2 Conioselinum anthriscoides “Chuanxiong” [Apiaceae; Chuanxiong radix et rhizoma]

*Conioselinum anthriscoides “Chuanxiong”* is the rhizome of the herbaceous plant *Ligusticum chuanxiong Hort.*, belonging to the Umbelliferae family. Due to its pharmacological properties of promoting blood circulation, resolving blood stasis, regulating qi, and relieving depression, it is widely utilized in the therapeutic management of various cardiovascular and cerebrovascular diseases. These therapeutic effects have been shown to be effective, and therefore, the medicinal use of *L. chuanxiong* has gained significant popularity. Pharmacological investigations have revealed its ability to dilate the coronary arteries, increase blood flow, and exhibit sedative and analgesic effects. The chemical composition of *C. anthriscoides “Chuanxiong”* is quite complex, mainly consisting of alkaloids, volatile oils, phthalides, organic acids, and lactones. Of particular interest is the active metabolite, tetramethylpyrazine, which has been found to possess significant vasorelaxant properties, antiplatelet aggregation effects, and the ability to maintain the structural and functional integrity of vascular endothelial cells. As a result, tetramethylpyrazine has been widely employed in the treatment of AS.

#### 1.2.1 Tetramethylpyrazine

Tetramethylpyrazine (TMP) is the principle bioactive metabolite of *C. anthriscoides “Chuanxiong,”* a traditional Chinese medicinal herb. A plethora of studies have demonstrated the remarkable efficacy of TMP in the treatment of AS, leading to its widespread clinical application in various ischemic conditions. Jiang F conducted a study that revealed the inhibitory effects of TMP on the progression of AS and hepatic lipid accumulation, thus providing the initial *in vivo* evidence supporting the anti-AS activity of TMP (Jiang et al., 2011). In another study by Sun MY, TMP intervention in mice with hyperlipidemia was found to alleviate endothelial dysfunction and mitigate inflammatory responses, demonstrating its therapeutic potential in the management of AS (Sun et al., 2018). Furthermore, Wang GF et al. conducted an experimental investigation involving the oral administration of TMP to rabbits with AS, and observed a significant reduction in the relative AS area, intima/media thickness ratio, and the number of monocytes within the intima (Wang et al., 2013). Additionally, a decrease in monocyte chemoattractant protein-1 and ICAM-1 levels in plasma, along with the inhibition of LOX (Lectin-like Oxidized-low-density Lipoprotein Receptor)-1 expression in rabbit aortas, was also noted. Through a comprehensive systematic review and meta-analysis encompassing 258 animal subjects from 12 studies, Li S ascertained that TMP exerts its anti-AS effects through anti-inflammatory activities and amelioration of lipid metabolism disorders in AS animal models (Li S. et al., 2022). Moreover, Ye T et al. unveiled the significant anti-inflammatory effects of TMP on human umbilical vein endothelial cells, thereby providing preliminary insights into the underlying mechanisms responsible for the anti-AS properties of TMP (Ye et al., 2021). Interestingly, Li XY discovered that TMP exerts its anti-AS effects by impeding the ERK, p38, and NF-κB signaling pathways, which consequently inhibits IL-8 expression in LPS-stimulated human umbilical vein endothelial cells, thus reinforcing its anti-AS activity (Li XY. et al., 2009).

#### 1.2.2 Ferulic acid

Ferulic acid is an active metabolite derived from *Angelica sinensis*, *Cimicifuga foetida*, *L. chuanxiong*, and other plants. Multiple studies have confirmed that ferulic acid can improve AS damage (Gu et al., 2021) and reduce AS plaque (Wu et al., 2022; Kwon et al., 2010). Chmielowski RA found that by downregulating scavenger receptors cluster of differentiation 36 (CD-36), Scavenger Receptor A1 (MSR-1), and LOX-1, nanoparticles containing ferulic acid significantly reduce oxLDL uptake and the potential of foam cell formation in macrophages (Chmielowski et al., 2017). Additionally, Wang CY has also demonstrated that by reducing endogenous reactive oxygen species generation, inflammation-related gene expression, and apoptosis levels, a novel formulation containing ferulic acid effectively reduces oxidative stress-induced injury in human umbilical vein endothelial cells (Wang CY. et al., 2020).

### 1.3 Coptis chinensis Franch [Ranunculaceae; Coptis chinensis radix et rhizoma]

*Coptis chinensis Franch*, originally documented in “Shen Nong’s Herbal Classic,” is classified as a superior herb. Belonging to the Ranunculaceae family, *C. chinensis* possesses a bitter taste and is renowned for its heat-clearing properties. It manifests efficacious effects in heat-clearing, dampness-drying, fire-purging, and detoxification. *Coptis chinensis Franch* contains diverse chemical metabolites, including coumarins, organic acids, alkaloids, sterols, flavonoids, and volatile oils. These metabolites confer antibacterial, anti-inflammatory, cholesterol-lowering, antioxidant, and hypoglycemic activities, thereby playing a pivotal role in regulating blood glucose and lipid levels, exerting anticancer effects, as well as preventing and ameliorating cardiovascular and cerebrovascular diseases.

#### 1.3.1 Berberine

Berberine, also known as berberine alkaloid, is the principal metabolite of *C. chinensis Franch*. In clinical settings, berberine has long been utilized as an over-the-counter medication for the treatment of diarrhea. With the progression of modern research, novel pharmacological properties of berberine have been consistently unearthed, revealing its diverse range of biological activities that showcase its efficacy in combating inflammation and AS. Numerous investigations have established the commendable anti-AS effects of berberine (Li X. et al., 2021; Shi et al., 2018; Li K. et al., 2009; Ma et al., 2021). Meta-analyses have consistently demonstrated the significant reduction in plaque area and foam cell content induced by berberine administration (Jia et al., 2022). Moreover, berberine has shown the ability to modulate the secretion of pro-inflammatory cytokines, further solidifying its therapeutic potential in AS management. The efficacy of berberine as an AS treatment lies in its capacity to regulate inflammation and plaque composition (Jia et al., 2022). Additional studies have elucidated that both high and low doses of berberine result in notable improvements in lipid profiles and systemic inflammation levels, thereby mitigating AS in ApoE^−/−^ mice exposed to high-fat diets (Wu et al., 2020). Zhu L et al. have provided evidence demonstrating the reduction of pro-inflammatory cytokines and chemokines in arterial and intestinal tissues as a result of berberine treatment, substantiating its efficacy in AS suppression (Zhu et al., 2018). Similarly, Man B observed improved lipid and glucose metabolism along with suppressed vascular inflammation in ApoE^−/−^ mice treated with berberine, consequently reducing AS development and plaque vulnerability (Man et al., 2022). Furthermore, a wealth of research has been dedicated to elucidating the mechanisms underlying the anti-AS effects of berberine. Song T et al. proposed that berberine achieves its beneficial effects through the regulation of the PI3K/AKT/mTOR signaling pathway, modulation of autophagy, promotion of cell proliferation, and inhibition of apoptosis, resulting in reduced lipid levels, improved intimal hyperplasia, and ameliorated carotid AS (Song and Chen, 2021). Wan Q et al. reported the preventive role of berberine in AS through the suppression of the p38MAPK and JNK signaling pathways (Wan et al., 2018). Similarly, Feng M’s investigations unveiled the substantial reduction in AS plaque area as a result of the anti-inflammatory properties of berberine in ApoE^−/−^ mice, which could be attributed to the inhibition of NF-κB translocation into the nucleus (Feng et al., 2017a). Zhang Peng et al. research has also confirmed the association with the NF-κB signaling pathway, demonstrating that berberine alleviates the progression of AS by regulating this pathway, both activating autophagy and reducing inflammation (Zhang P. et al., 2025). Moreover, berberine treatment induced autophagy and inhibited inflammation in J774A.1 cells through the activation of the AMPK/mTOR signaling pathway. Notably, this study provides novel insights into the molecular mechanisms and therapeutic potential of berberine in AS treatment (Fan et al., 2015). Additionally, Jiang Y’s findings highlight the significance of berberine in alleviating NLRP3 inflammasome activation by reducing IL-1β secretion from macrophages, thereby identifying it as a crucial therapeutic target for AS management (Jiang et al., 2017). Zheng Yinghong’s findings suggest the potential of berberine as a novel approach to addressing the senescence-associated secretory phenotypes-associated inflammation through RXRα/PPARγ/NEDD4 Pathway in atherosclerosis (Zheng et al., 2025). Another significant study demonstrates that avocado-derived extracellular vesicles loaded with ginkgetin and berberine mitigate inflammation and suppress macrophage foam cell formation. This dual action attenuates atherosclerosis progression while leveraging the synergistic potential of natural metabolites like berberine (Sharma et al., 2024).

#### 1.3.2 Coptisine

Coptisine, existing in the form of a quaternary ammonium salt, is an isoquinoline alkaloid. Its content is considered one of the quality control standards for *Coptis* in the Chinese Pharmacopoeia (2015 edition). It possesses notable pharmacological effects, including antibacterial, anti-inflammatory, hypoglycemic, and lipid-lowering activities. Due to its lipid-lowering and anti-inflammatory properties, coptisine has been shown to exert beneficial effects in the treatment of AS. Coptisine demonstrates the ability to reduce the levels of pro-inflammatory cytokines such as IL-6 in serum, as well as decrease the area of primary AS plaques. These anti-AS effects are attributed to the inhibition of the MAPK signaling pathway activation and translocation of NF-κB (Feng et al., 2017b). Additionally, coptisine can effectively suppress LPS-induced inflammation in macrophages through the blockade of NF-κB, MAPK, and PI3K/Akt activation. Its potential as a therapeutic agent for the prevention and treatment of inflammatory diseases, including AS, has been evidenced (Wu et al., 2016).

### 1.4 Panax notoginseng (Burkill) F.H.Chen [Araliaceae; Panax notoginseng radix et rhizoma]

This product consists of the dried roots and rhizomes of Panax notoginseng, a plant belonging to the Araliaceae family. *Panax notoginseng (Burkill) F.H.Chen* contains various saponins, with a total saponin content ranging from 8% to 12%. These saponins, similar to ginsenosides, belong to the dammarane-type tetracyclic triterpenoid saponins. Among them, ginsenosides Rg1, Rb1, and notoginsenoside R1 are the major metabolites and quality control markers for *P. notoginseng (Burkill) F.H.Chen* as listed in the Chinese Pharmacopoeia. Additionally, *P. notoginseng (Burkill) F.H.Chen* also contains a small amount of quercetin. The pharmacological properties of *P. notoginseng (Burkill) F.H.Chen* include promoting blood circulation, reducing inflammation and pain, improving vascular patency, inhibiting platelet aggregation, and increasing cerebral blood flow. These properties contribute to its preventive and therapeutic effects on cardiovascular diseases.

#### 1.4.1 Notoginsenoside R1

Notoginsenoside R1 is one of the major active metabolites of the total saponins found in *P. notoginseng (Burkill) F.H.Chen* (the other major metabolite, ginsenosides, will be discussed in the ginseng section). Notoginsenoside R1 exerts beneficial effects on various cardiovascular diseases, particularly AS-related conditions. It achieves this through anti-inflammatory and anti-apoptotic mechanisms, modulation of energy metabolism, inhibition of oxidative stress, suppression of myocardial fibrosis, antiarrhythmic effects, vasodilation, and promotion of angiogenesis. Animal experiments have demonstrated the anti-AS effects of notoginsenoside R1, accompanied by a reduction in pro-inflammatory cytokines levels, such as IL-2 (Jia et al., 2014). Additionally, several studies have reported that notoginsenoside R1 can prevent ox-LDL-induced apoptosis of human umbilical vein endothelial cells and inhibit the AS response induced by ox-LDL (Zhao et al., 2020a; Zhu et al., 2020; Fu et al., 2018). Notably, Liu D’s findings reveal that notoginsenoside R1 diminishes the secretion of inflammatory factors in AS mice, enhances autophagy, ameliorates aortic aging, and ultimately reduces AS plaques. These effects are likely achieved by efficaciously activating the AMPK pathway to enhance autophagy and suppress senescence (Liu et al., 2023). Notoginsenoside R1 significantly modulates blood lipid profiles and suppresses pro-inflammatory cytokines, thereby attenuating the progression of atherosclerosis. This effect is likely mediated through suppression of the NLRP3/cleaved caspase-1/IL-1β inflammatory axis and downregulation of the JNK2/p38 MAPK/VEGF pathway implicated in endothelial damage (Ma et al., 2024). Furthermore, the XIST/miR-221-3p/TRAF6 axis and the TLR4/NF-κB signaling pathway have been suggested as plausible targets for the anti-AS effects of notoginsenoside R1. It is noteworthy that the combination of notoginsenoside R1 and saikosaponin B2 synergistically activates macrophage autophagy by suppressing the PI3K/AKT/mTOR pathway, thereby attenuating lipid accumulation, inflammation, and apoptosis in atherosclerotic models (Wang et al., 2025).

#### 1.4.2 Ginsenoside Rg1

Ginsenoside Rg1, a major active component found in *P. notoginseng (Burkill) F.H.Chen* and ginseng, belongs to the class of triterpenoid saponin metabolites and has emerged as a prominent subject of research in the field of individual ginsenosides in recent years. Ginsenoside Rg1 exerts significant effects on cardiac function, cardiovascular health, and blood circulation. A study conducted by Yang P revealed that ginsenoside Rg1 exhibits the potential to suppress macrophage apoptosis by activating the AMPK/mTOR signaling pathway, thereby providing compelling evidence for its use in the treatment of AS (Yang P. et al., 2018).

#### 1.4.3 Quercetin

Quercetin, also known as meletin or quercetin flavonoids, is a naturally occurring flavonoid metabolite that has been proven to possess therapeutic efficacy in the treatment of various diseases, such as hypertension, inflammation, and vascular disorders. Its pharmacological value, particularly in treating cardiovascular diseases, is significant and holds substantial potential. Numerous studies have established that quercetin can modulate lipid metabolism and mitigate inflammatory responses, thereby exerting inhibitory effects on the formation of AS plaques (Garelnabi et al., 2014; Juźwiak et al., 2005; Bhaskar et al., 2013; Jiang et al., 2020). In an experimental investigation conducted by Li SS, quercetin intervention in ApoE^−/−^ mice subjected to a high-fat diet resulted in a marked reduction in AS plaque area as well as significant downregulation of Proprotein Convertase Cubtilisin/Kexin Type 9 (PCSK9), TNF-α, and IL-6 expression (Li et al., 2020). Cao H demonstrated that quercetin impedes the progression of AS through its ability to regulate autophagy in RAW264.7 foam cells induced by oxLDL, leading to diminished pro-inflammatory cytokines levels (Cao et al., 2019). Jia Q’s findings indicated that quercetin prevents the development of AS in ApoE^−/−^ mice by modulating the expression of PCSK9, CD36, Peroxisome Proliferator-activated Receptor γ (PPARγ), Liver X Receptor α (LXRα), and ATP Binding Cassette Transporter A1, while concurrently reducing the levels of pro-inflammatory cytokines including TNF-α and IL-6 (Jia et al., 2019). Tsai CF’s study revealed that quercetin brings about an anti-AS effect by reducing the expression levels of M1 markers, such as IL-6, TNF-α, and IL-1β, in both macrophages and microglial cells (Tsai et al., 2021). Moreover, the research conducted by Li H demonstrated that quercetin significantly inhibits the activation of the NLRP3 inflammasome in macrophages loaded with oxLDL. This mechanism subsequently alleviates cellular lipid degeneration and IL-1β secretion, thereby exhibiting preventive and therapeutic effects against AS (Li H. et al., 2021). Zhang F’s investigations revealed that quercetin improves the pathological physiology of AS in rat carotid arteries by modulating the AMPK/SIRT1/NF-κB signaling pathway, thereby suppressing oxidative stress and inflammatory responses (Zhang et al., 2020). These findings are corroborated by a recent study by Wang Yu-Man, which revealed that quercetin reduces the expression of inflammatory cytokines, NLRP3 inflammasome activity, and NF-κB activation in endothelial cells. The underlying mechanism involves the attenuation of atherosclerosis through the suppression of inflammation, achieved by inhibiting the Pio 1-mediated NF-κB/IL-1β and NLRP3/caspase-1/IL-1β signaling axes (Wang et al., 2024). Additionally, Lu XL’s study demonstrated that quercetin, through modulation of the ROS-regulated PI3K/AKT signaling pathway, inhibits inflammation and apoptosis, effectively ameliorating high-fructose-induced AS (Lu et al., 2017).

### 1.5 Scutellaria baicalensis Georgi [Lamiaceae; Scutellaria baicalensis dried root]

This product consists of the dried roots of Scutellaria baicalensis Georgi, a member of the Lamiaceae family. *Scutellaria baicalensis Georgi* is rich in flavonoid constituents, with more than 40 flavonoids identified, including baicalin, baicalein, wogonin, wogonoside, and oroxylin A. Extensive pharmacological studies have revealed a diverse range of effects of *S. baicalensis Georgi*, including anti-inflammatory, anti-tumor, antioxidant, hepatorenal protective, and maintenance of cardiovascular and cerebrovascular function. Consequently, it has found broad clinical applications. Notably, natural metabolites such as baicalin, baicalein, and wogonin, possessing anti-inflammatory properties, have been widely employed in the prevention and treatment of AS.

#### 1.5.1 Baicalin

Baicalin is a major flavonoid metabolite extracted and isolated from the rhizome of Scutellaria baicalensis Georgi. Research reports have demonstrated that baicalin exhibits significant therapeutic efficacy in the treatment of AS. Mechanistically, baicalin has been shown to modulate the expression of AS-associated inflammatory factors, thereby ameliorating endothelial cell injury and maintaining endothelial homeostasis, ultimately exerting an anti-AS effect. Notably, studies conducted by Liao P have indicated that treatment with baicalin in ApoE^−/−^ mice exposed to a high cholesterol diet leads to significant reduction of AS plaque area within the aorta (Liao et al., 2014). Numerous investigations have explored the anti-AS mechanisms of baicalin and have demonstrated its capacity to suppress the NF-κB and p38MAPK signaling pathways, consequently diminishing the levels of AS-induced pro-inflammatory cytokines in the serum of ApoE^−/−^ mice, thereby alleviating AS (Wu et al., 2018; Yang et al., 2025). Zhao J’s study has provided evidence showing that baicalin downregulates the expression and activation of the NLRP3 inflammasome, resulting in decreased production of pro-inflammatory cytokines, mitochondrial reactive oxygen species, total reactive oxygen species, ICAM-1, and VCAM-1, ultimately leading to reduced plaque area and exerting its anti-AS effects (Zhao et al., 2020b). Of particular significance, baicalin is recognized for its protective effect against thrombin-induced injury in human umbilical vein endothelial cells. This protective mechanism may be mediated through inhibition of protease activated receptors-1 expression and consequent NF-κB activation via ERK1/2 signaling pathway (Zhang et al., 2019). *In vitro* experiments have additionally demonstrated that baicalin can upregulate the expression of miR-126-5p by targeting high-mobility group box 1, thereby inhibiting the proliferation and migration of ox-LDL-VSMCs and subsequently preventing AS (Chen Z. et al., 2019). Furthermore, Wang B’s study has revealed that baicalin exerts its anti-inflammatory effects and potentially prevents AS lesions by enhancing Wnt1 expression while inhibiting dickkopf-related protein-1 expression (Wang et al., 2016).

#### 1.5.2 Baicalein

Baicalein is a flavonoid metabolite with a wide range of biological effects. It has been widely used in the prevention and treatment of cardiovascular and cerebrovascular diseases for a considerable period of time, displaying favorable regulation of inflammatory responses. Numerous *in vivo* and *in vitro* studies have provided evidence supporting the robust anti-AS activity of baicalein (Chan et al., 2016; Zhang et al., 2015; Song et al., 2014). Lipid accumulation and inflammatory reactions are recognized as two primary risk factors associated with AS. Baicalin exerts beneficial effects on lipid accumulation and inflammatory reactions within macrophages by activating the PPARγ/LXRα signaling pathway, thus demonstrating its protective activity against AS (Zhang et al., 2022). The results of Zhang X’s investigations suggest that baicalein specifically intervenes in the inflammation-related AMPK//Mfn-2/MAPKs signaling pathway, leading to a decline in the levels of pro-inflammatory cytokines, thereby exerting notable anti-AS effects (Zhang et al., 2021). Additionally, *in vitro* studies have revealed that baicalein counteracts oxidative stress and inflammation induced by oxLDL through the regulation of AMPK-α (Tsai et al., 2016).

#### 1.5.3 Wogonin

Wogonin is a flavonoid metabolite isolated from the roots of Scutellaria baicalensis Georgi. It has exhibited a range of biological activities, including anti-inflammatory, anti-tumor, cardioprotective, and neuroprotective effects. Consequently, it is frequently employed in the treatment of inflammatory diseases, AS, and hyperlipidemia. The vascular inflammatory process is considered to play a pivotal role in the initiation and progression of AS. Notably, studies conducted by Ku SK have demonstrated wogonin’s capacity to inhibit the vascular inflammatory process in both human umbilical vein endothelial cells and mice, induced by high glucose conditions (Ku and Bae, 2015). This finding underscores the metabolite’s significant therapeutic benefits, specifically in mitigating diabetic complications and AS. Moreover, wogonin exerts its preventive and therapeutic effects on AS by interfering with the DAG-PKC pathway and ameliorating vascular smooth muscle cell apoptosis induced by lipotoxicity (Liu et al., 2011). It is worth noting that, a recent study demonstrated that wogonin reprograms macrophage metabolism from glycolysis to fatty acid oxidation through activation of the PPARα-KLF11-YAP1 pathway, thereby reducing inflammation and foam cell formation, ultimately attenuating atherogenesis. This study conclusively demonstrates that baicalein exerts anti-atherosclerotic effects by dually modulating blood lipids and inflammation (Ma et al., 2025).

### 1.6 Pueraria montana var. lobata (Willd.) Maesen and S.M.Almeida ex Sanjappa and Predeep [Fabaceae; Pueraria montana dried root]

This product consists of the dried roots of Puerarialobata (Willd.) Ohwi, a leguminous plant commonly known as wild kudzu. *Pueraria montana* var. *lobata (Willd.) Maesen and S.M.Almeida ex Sanjappa and Predeep* is predominantly comprised of flavonoids and coumarins, which exhibit notable pharmacological properties, including antipyretic, anti-inflammatory, anti-infective, antihypertensive, antihyperglycemic, hypolipidemic, and reproductive function-enhancing effects. In clinical practice, *P. montana* var. *lobata (Willd.) Maesen and S.M.Almeida ex Sanjappa and Predeep* is frequently utilized in the form of complex traditional Chinese medicine formulations for the management of diabetes, gastrointestinal disorders, cardiovascular diseases, and related conditions. Notably, puerarin constitutes the major metabolite of flavonoid components.

#### 1.6.1 Puerarin

Puerarin is an isoflavone metabolite extracted from the root of Puerarialobata, which has been employed in traditional Chinese medicine for thousands of years. Extensive experimental and clinical evidence has demonstrated the significant therapeutic potential of puerarin in the treatment of various cardiovascular diseases, including angina, myocardial ischemia-reperfusion injury, hypertension, diabetes, and others. Clinical studies have established that puerarin effectively inhibits the progression of intimal thickening in rheumatoid arthritis of the carotid artery (Yang M. et al., 2018). Animal experiments have further supported these findings by illustrating puerarin’s ability to suppress the formation and development of AS plaques, as well as inhibit the migration and proliferation of vascular smooth muscle cells, thus highlighting its promising role in cardiovascular disease prevention and treatment (Bao et al., 2015). Notably, in ApoE^−/−^ mice with AS, puerarin has demonstrated its capacity to reduce the expression of pro-inflammatory cytokines, which are critical markers of AS. This therapeutic effect is believed to be mediated through the miR-29b-3p/IGF1 pathway (Li et al., 2023). And Li Ye-Ting’s study demonstrated that puerarin attenuates acrolein-induced AS and reduces inflammation by suppressing HDAC1-mediated oxidative stress through modulation of the JNK pathway (Li et al., 2024). Deng Y’s research discovered that puerarin inhibits monocyte adhesion *in vitro* and *in vivo*, reduces the expression of inflammatory factors such as IL-8, and alleviates AS lesions in ApoE^−/−^ mice by activating the ERK5/KLF2 signaling pathway (Deng et al., 2018). Impaired endothelial cell function induced by oxLDL is considered the first step in AS development. Bao MH’s study found that puerarin inhibits oxLDL-induced endothelial cell injury by suppressing LOX-1-mediated p38MAPK-NF-κB inflammation and PKB-eNOS signaling pathways (Bao et al., 2014). Additionally, the experimental research conducted by Ji et al. (2016) and the clinical study by Yang et al. (2010) provide further evidence that the anti-inflammatory effects of puerarin exerted through inhibition of the NF-κB signaling pathway in the treatment of AS. Last but not least, nanoparticle-mediated delivery of puerarin significantly enhances its pharmacokinetic profile, leading to potent attenuation of oxidative stress and inflammation. These effects collectively contribute to plaque stabilization and retardation of atherosclerotic disease progression (Liu et al., 2025).

### 1.7 Panax ginseng C.A.Mey. [Araliaceae; Panax ginseng dried root]

*Panax ginseng C.A.Mey*, a medicinal herb indigenous to the northeastern region of China, is derived from the dried roots and rhizomes of the Araliaceae plant Panax ginseng C. A. Mey. Renowned as a traditional and esteemed Chinese herbal medicine, its long-term usage has been associated with remarkable attributes such as anti-aging properties and revitalization of vital energy. Consequently, *P. ginseng C.A.Mey* has garnered extensive attention in clinical applications. The continuous advancement of pharmaceutical analytical techniques has facilitated the discovery that ginseng manifests a broad spectrum of biological activities and exhibits a complex array of chemical metabolites. Notably, considerable progress has been made in the identification and classification of its active components, mainly comprising ginsenosides, which can be further subcategorized into Ra1-3, Rb1-3, Rc, Rd, Rg3, Re, Rf, Rg1, Rg2, Rh1, Ro, and so forth based on their ginsenoside structures. The bioactive metabolites of *P. ginseng C.A.Mey* demonstrate various therapeutic effects such as anti-inflammatory, antioxidative, mitochondrial protection, and calcium overload inhibition, showing potential for the prevention and treatment of AS-related diseases.

#### 1.7.1 Ginsenoside Rb1

Ginsenoside Rb1 is a monomeric triterpene glycoside found in *P. ginseng C.A.Mey*, possessing a diverse range of biological activities. Among its various biological functions, the anti-inflammatory effect is considered to be a prominent characteristic of ginsenoside Rb1. Both preclinical and clinical evidence suggest that ginsenoside Rb1 exerts anti-inflammatory actions in diseases associated with the central nervous system, as well as the cardiovascular system. By inhibiting the levels of pro-inflammatory cytokines in the bloodstream and attenuating apoptosis related to anti-inflammatory activity, ginsenoside Rb1 effectively suppresses the early onset of AS in ApoE^−/−^ mice (Zhou et al., 2018). Several studies have demonstrated the ability of Rb1 to enhance the stability of AS plaques (Yang et al., 2021; Qiao et al., 2017; Zhang et al., 2018). Notably, Yang X’s research has shown that the inhibitory effects of ginsenoside Rb1 on plaque growth and the promotion of plaque stability are attributed to its ability to suppress neovascularization and inflammation (Yang et al., 2021). Moreover, Qiao L’s investigation revealed that ginsenoside Rb1 promotes plaque stability by improving macrophage foam cell autophagy and lipid metabolism (Qiao et al., 2017). Additionally, Zhang X’s study found that ginsenoside Rb1 enhances plaque stability by facilitating the polarization of anti-inflammatory M2 macrophages and increasing the production of IL-4 and/or IL-13 (Zhang et al., 2018). Considering that endothelial cell damage serves as an initial trigger for AS, research has demonstrated that ginsenoside Rb1 effectively mitigates TNF-α-induced endothelial cell inflammation by inhibiting the NF-κB, JNK, and p38 signaling pathways, thereby reducing the levels of pro-inflammatory cytokines (Zhou et al., 2017). Furthermore, Yang G’s investigation elucidated that ginsenoside Rb1’s anti-AS potential is mediated through the activation of the GPER-mediated PI3K/Akt signaling pathway, thus inhibiting endothelial cell apoptosis and the production of pro-inflammatory cytokines, while restoring normal endothelial cell functionality (Yang et al., 2019). With the development of medical engineering integration and the concept of “integration of Chinese and Western medicine”, Biomimetic Ginsenoside Rb1 and Probucol Co-Assembled Nanoparticles effectively retarded atherosclerotic plaque formation through synergistic effects of antioxidative stress, anti-inflammation, and inhibition of lipid deposition.

#### 1.7.2 Ginsenoside Rh1

Ginsenoside Rh1 is a triterpene glycoside of the dammarane type found in *P. ginseng C.A.Mey*. Despite its relatively low abundance in *P. ginseng C.A.Mey*, it possesses potent pharmacological activity, including significant anti-cancer and anti-tumor effects, as well as notable efficacy in preventing coronary heart disease. Dysfunction of endothelial cells plays a crucial role in the initiation of AS, with the induction of inflammatory cytokines and adhesion molecules being key factors. Research findings indicate that ginsenoside Rh1 effectively inhibits lipopolysaccharide-induced inflammation and apoptosis in endothelial cells by suppressing the STAT3/NF-κB and endoplasmic reticulum stress signaling pathways. Consequently, it effectively attenuates endothelial cell dysfunction (Jin et al., 2022).

#### 1.7.3 Ginsenoside Rg2

Ginsenoside Rg2 is one of the predominant and most significant bioactive metabolites found in *P. ginseng C.A.Mey*, exhibiting a richness in content. It possesses diverse pharmacological activities, including immunomodulatory functions and anti-inflammatory properties, which render it extensively employed in the realm of cardiovascular and cerebrovascular diseases. The anti-AS effects of ginsenoside Rg2 are demonstrated not only at the cellular level but also in animal models, as it modulates inflammatory signaling pathways, such as NF-κB and p-ERK (Xue et al., 2023). Notably, research conducted by Cho YS reveals that ginsenoside Rg2 showcases an inhibitory impact on the expression of adhesion molecules in human umbilical vein endothelial cells stimulated by lipopolysaccharide, therefore contributing to safeguarding against vascular inflammatory diseases (Cho et al., 2013).

#### 1.7.4 Ginsenoside Rg3

Ginsenoside Rg3, the primary active metabolite found in *P. ginseng C.A.Mey*, possesses a range of therapeutic effects, such as anti-tumor, anti-cardiovascular disease, anti-inflammatory, anti-fatigue, and neuroprotective properties. PPARγ, a critical nuclear transcription factor involved in inflammation and macrophage differentiation, has been identified by Geng J as a target for the regulatory role of ginsenoside Rg3 (Geng et al., 2020). This regulation mitigates oxLDL-induced endothelial dysfunction and prevents the onset of AS in ApoE^−/−^ mice by modulating the PPARγ/FAK signaling pathway. Furthermore, Guo M’s research has substantiated that ginsenoside Rg3 enhances the stability of AS lesions and reduces plaque burden. This effect is concurrent with an increase in M2 macrophages and a decrease in M1 macrophages, suggesting that the protective impact on endothelial cells and inhibition of AS may be mediated via the PPARγ pathway (Guo et al., 2018).

### 1.8 Angelica sinensis (Oliv.) Diels [Apiaceae; Angelica sinensis dried root]

This product comprises the dried roots of Angelicasinensis (Oliv.) Diels, a member of the Umbelliferae family. It contains volatile oil and water-soluble metabolites. The principal metabolite of the volatile oil is ligustilide, while the water-soluble fraction contains ferulic acid (discussed in *L. chuanxiong*). Additionally, it contains *A. sinensis (Oliv.) Diels* polysaccharide among other metabolites. It exerts notable pharmacological effects such as anti-inflammatory, hepatoprotective, immunomodulatory, cardiovascular regulatory, and bronchodilatory activities, playing a pivotal role in safeguarding the cardiovascular system and mitigating the risk of coronary heart disease and AS.

#### 1.8.1 Ligustilide

Ligustilide, an active metabolite present in elevated quantities in the volatile oil of *A. sinensis (Oliv.) Diels*, is associated with diverse pharmacological activities, including anti-inflammatory, analgesic, antioxidant, and neuroprotective effects. These activities render it valuable for the prevention and treatment of cardiovascular and cerebrovascular disorders. An investigation employing network pharmacology recently revealed new insights into ligustilide’s anti-AS effects, uncovering its capacity to attenuate inflammation, and hinder cellular proliferation and migration. As a result, ligustilide may serve as a prospective therapeutic target for AS treatment (Fang et al., 2022). Chronic vascular inflammation, which occurs within the vascular endothelium, is a central driver of cardiovascular pathogenesis. Encouragingly, Choi ES’ research discovered that ligustilide significantly suppressed TNF-α-induced ROS production and activated the NF-κB signaling pathway. Consequently, ligustilide demonstrates potential in alleviating vascular inflammation and impeding AS progression (Choi et al., 2018).

#### 1.8.2 Angelica sinensis polysaccharides

Angelica sinensis polysaccharides, a natural high-molecular-weight metabolite, constitute the primary bioactive components of *A. sinensis (Oliv.) Diels*. These polysaccharides possess diverse pharmacological effects including hematopoietic promotion, immune enhancement, anti-tumor properties, and antioxidant activity. The extensive pharmacological potential of Angelica sinensis polysaccharides is closely intertwined with their specific structural characteristics. Through a network pharmacology study, Niu X identified the cardioprotective metabolite of Angelica sinensis as Angelica sinensis polysaccharides. Subsequent experimental validation in animal models demonstrated the capacity of Angelica sinensis polysaccharides to reduce infarct size and preserve cardiac function in acute myocardial infarction-induced rats. This effect is likely mediated through AMPK-PGC1α pathway activation and subsequent modulation of pro-inflammatory cytokine levels (Niu et al., 2018).

### 1.9 Rhodiola rosea L. [Crassulaceae; Rhodiola dried roots and rhizomes ]

*Rhodiola* is the dried root and rhizome of the plant Rhodiola rosea L., which belongs to the Crassulaceae family. It thrives in high-altitude, cold, and ultraviolet radiation-rich environments, earning it the reputation of being the “Tibetan ginseng” and the “Eastern panacea.” For over a millennium, it has been utilized in traditional medicine across Asian countries. Its chemical composition comprises mainly of salidroside, quercetin, tyrosol, cinnamyl alcohol, rhodiocyanoside, polysaccharides, and several others. Notably, it exhibits pharmacological properties such as anti-inflammatory, antioxidant, anti-fatigue, anti-hypoxia, and anticancer activities.

#### 1.9.1 Salidroside

Currently, over forty chemical components have been isolated from *Rhodiola rosea L.*, with salidroside being its main active metabolite and one of the markers used to assess the quality of *R. rosea L.* medicinal materials. It possesses pharmacological activities such as anti-inflammatory, antioxidant, antitumor, and antiviral effects, providing protective effects on cardiovascular, nervous, renal, and hepatic systems. Numerous *in vitro* and *in vivo* studies have confirmed the effective improvement of AS by salidroside (Li L. et al., 2021; Bai et al., 2020; Zhu et al., 2019a; Ni et al., 2017; Xing et al., 2015). Research has demonstrated that salidroside can increase mitochondrial membrane potential, reduce ROS levels, and inhibit NF-κB activation, thereby modulating the expression of pro-inflammatory factors and mitochondrial homeostasis-related proteins, suppressing M1 macrophage polarization, and maintaining mitochondrial homeostasis after macrophage polarization, ultimately exerting an anti-AS effect (Wang et al., 2023). Endothelial cell senescence is a significant cause of AS. Salidroside reduces the expression of inflammatory cytokines and upregulates the expression of SIRT3 to alleviate endothelial cell senescence (Xing et al., 2018). Studies by Xing SS have found that salidroside reduces plaque formation in AS by inhibiting endothelial cell apoptosis, suppressing caspase-1 activation and IL-1β release, and decreasing Gasdermin D expression (Xing et al., 2020). Li R’s study revealed that salidroside reduces vascular inflammation by mediating the activation of MAPK and NF-κB in TNF-α-induced endothelial cells, showing potential value in anti-AS therapy (Li et al., 2019).

### 1.10 Astragalus mongholicus Bunge [Fabaceae; Astragalus mongholicus dried root]

*Radix Astragali* is derived from the dried roots of *Astragalus mongholicus Bunge*, which are leguminous plants of the Fabaceae family. Modern research has identified that *Radix Astragali* primarily consists of flavonoids, saponins, and polysaccharides. *Radix Astragali* exhibits diverse pharmacological activities, including potent anti-inflammatory properties, antioxidant effects, and the ability to inhibit endothelial monolayer permeability in blood vessels. Notably, it exerts protective effects on the cardiovascular and cerebrovascular systems, enhances immune responses, and shows promising anti-tumor effects.

#### 1.10.1 Astragaloside

Saponins, the primary active metabolites of *Radix Astragali*, exhibit pharmacological properties including anti-inflammatory, immunomodulatory, antioxidant, and anti-apoptotic effects. Among these metabolites, Astragaloside, a significant saponin, finds extensive application in cardiovascular diseases. Qin HW et al. employed cellular and animal experiments to demonstrate that Astragaloside effectively reduces the levels of pro-inflammatory cytokines, thereby suppressing vascular inflammation and ameliorating endothelial cell injury. Consequently, it plays a pivotal role in preventing the onset and progression of AS. The underlying mechanism involves the targeted regulation of miR-17-5p, thereby impacting the PCSK9/VLDLR signaling pathway (Qin et al., 2022). Endothelial injury caused by oxLDL constitutes a major mechanism of AS. Research findings indicate that Astragaloside exerts protective effects by inhibiting apoptosis and inflammation, while simultaneously restoring the vitality and migratory capability of oxLDL-induced human umbilical vein endothelial cells (Shao et al., 2021). Similar conclusions were drawn from studies conducted by Chen et al. (2023), Tian et al. (2022), Zhu et al. (2019b), confirming the ability of Astragaloside to reduce apoptosis, inflammation, and autophagy, thereby diminishing the levels of pro-inflammatory cytokines. Moreover, it safeguards human umbilical vein endothelial cells against oxLDL-induced damage, thereby enhancing the stability of AS plaques.

### 1.11 Discussion

**FIGURE 1:**
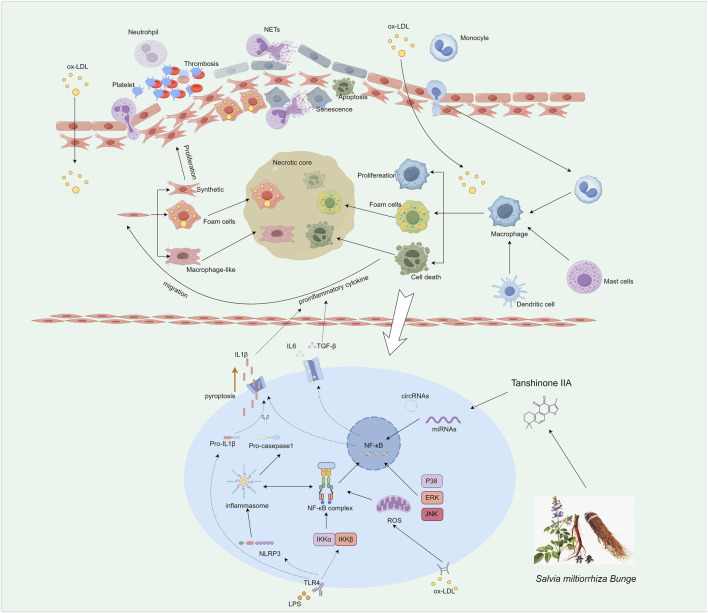
Pathogenesis of AS Driven by Inflammatory Responses. The initial step involves endothelial dysfunction and platelet activation. Subsequently, recruited monocytes differentiate into macrophages, which internalize ox-LDL to become lipid-laden foam cells, a hallmark of early lesions. Vascular smooth muscle cells migrate from the media, proliferate, and undergo phenotypic switching, contributing to fibrous cap formation and foam cell generation. In advanced stages, sustained inflammation is mediated by macrophages via pathways such as NLRP3 inflammasome activation and regulation by non-coding RNAs, leading to prolific pro-inflammatory cytokine release and plaque progression.

The formation of AS involves complex inflammatory processes, and endothelial cell injury plays a critical role in the development of this condition. Maintaining the integrity of endothelial cells is of utmost importance. Numerous researchers have shown great interest in the use of natural herbal products, which contain various active monomers or effective metabolites that exhibit promising anti-AS effects. In this review, we conducted a comprehensive analysis of the current research progress in utilizing active components of traditional Chinese medicine for regulating inflammation in order to combat AS, as shown in Supplementary Table S1; Figure 1. We found that natural products with anti-AS properties are primarily derived from plants belonging to the families Araliaceae, Umbelliferae, Fabaceae, and Lamiaceae. The chemical structures of these natural products mainly consist of flavonoids, alkaloids, saponins, and polysaccharides, among others. This suggests that natural products with different structural types can exhibit anti-AS activities. However, flavonoids, alkaloids, and saponins show greater potential, for example, Flavonoids exemplified by Baicalin (Significantly reducing plaque), Alkaloids including Berberine (Regulating inflammation and plaque composition)and Saponins such as Notoginsenoside R1 (Significantly modulating blood lipid profiles and suppresses pro-inflammatory cytokines) and thus deserve prioritization in future drug development.

We have also observed that the research frontier on the anti-inflammatory effects of plant-derived natural products against AS is undergoing significant deepening and expansion. The core shift involves moving beyond the suppression of individual inflammatory factors (e.g., TNF-α, IL-6) towards targeting the regulation of complex inflammatory networks and their upstream signaling hubs. Regarding novel mechanisms, targeting NLRP3 inflammasome activation and its mediated pyroptosis has emerged as a major focus. For instance, salidroside inhibits Gasdermin D expression and the caspase-1/IL-1β pathway, while tanshinone IIA and coptisine have also been demonstrated to effectively suppress NLRP3. The gut microbiota-host immune crosstalk axis is increasingly recognized as a pivotal novel target. Metabolites like berberine attenuate systemic low-grade inflammation and vascular pathology indirectly by reshaping microbial community structure, reducing pro-inflammatory metabolites, or enhancing barrier function. Non-coding RNAs (e.g., miRNAs, circRNAs) are widely revealed as crucial regulatory mediators. Examples include tanshinone IIA modulating macrophage autophagy and polarization via miR-375/KLF4, puerarin inhibiting vascular smooth muscle cell inflammation via miR-29b-3p/IGF1. Emerging trends emphasize precise cell-specific interventions (e.g., macrophage M1/M2 polarization, endothelial cell senescence/pyroptosis, vascular smooth muscle cell phenotypic switching) and exploring how natural products synergistically regulate multiple pathological processes—such as inflammation, autophagy, and lipid metabolism—through core signaling nodes (e.g., AMPK/PI3K/Akt, PPARγ/LXRα). These discoveries provide critically important theoretical foundations and innovative perspectives for developing novel, natural product-based anti-AS therapeutics with enhanced selectivity and multi-target synergistic actions.

Undeniably, owing to their advantages such as multiple targets and rich diversity, natural products with abundant resources will become an important source of anti-AS drugs. Nevertheless, the existing research still has several limitations. For instance, the efficacy of many natural products remains unclear or requires further improvement. In some cases, the efficacy of certain natural products lacks support from clinical trial data. Moreover, although several natural products have demonstrated promising anti-AS effects in cell and animal experiments, their clinical effectiveness is not ideal, and some have even yielded negative results in clinical trials. Furthermore, mechanistic studies of various natural products remain incomplete and lack in-depth exploration. In addition, for those natural products that have shown favorable outcomes in clinical trials, there is a need for more downstream research, such as systematic evaluations of drug safety, clarification of adverse reactions, and drug interactions, and the understanding of appropriate dosage and administration regimens. Additionally, natural products face challenges related to low raw material content, low bioavailability, and other issues. And batch variability in herbal extracts is another barrier for clinical translation. Although plant metabolites show considerable promise in anti-atherosclerosis research, their activity assessment requires heightened vigilance against systematic bias introduced by Pan-Assay Interference Compounds (PAINS). PAINS represent a class of compounds that generate false-positive signals in diverse *in vitro* assays through non-specific mechanisms, including redox cycling, protein aggregation, fluorescence quenching/enhancement, or metal chelation. Common structural classes in plant secondary metabolites—such as polyphenols, quinones, and terpenoids—have been demonstrated to carry elevated PAINS risk (Magalhães et al., 2021). While these compounds may exhibit apparent high activity in cell-based or enzyme-targeted *in vitro* models, their effects frequently lack target specificity and rarely translate into *in vivo* pharmacological effects or clinical value (Bolz et al., 2021).

Therefore, building upon previous research, future endeavors should focus on two aspects. Firstly, it is crucial to further explore a greater variety of natural product resources and conduct structural modifications and optimizations based on identified natural products. This approach will yield a broader range of candidate metabolites with enhanced activity and increased bioavailability, thus facilitating the development of novel, safe, and effective anti-AS drugs. Secondly, it is imperative to continue refining the mechanistic understanding of various anti-AS natural products already discovered. Subsequently, extensive clinical trials must be conducted to validate their efficacy and evaluate their safety. Such efforts will actively promote their clinical application and industrial production, leading to the development of anti-AS medications with superior therapeutic effects and reduced adverse reactions, ultimately contributing towards the prevention of cardiovascular diseases triggered by AS.

In summary, we hope that this comprehensive review will provide valuable insights into the potential and pharmacological mechanisms of current natural medicines, thereby offering support for future drug discoveries.
