# Supplementary material for: Plant-derived natural products targeting inflammation in treatment of atherosclerosis
Source: Front Pharmacol. 2025 Oct 2;16:1642183. doi: 10.3389/fphar.2025.1642183 (PMC12528052; doi:10.3389/fphar.2025.1642183)
Supplement: Supplementary file 1 [file Table1.pdf]

Supplementary Material

Supplementary Table 1 Anti-inflammation effects of Plant-Derived Natural Products and the underlying molecular mechanism.

TCM	Constituent	Chemical formula	Molecular formula	Study type	Subjects	Dose	Potential mechanism	References
Salvia miltiorrhiza	Tan IIA	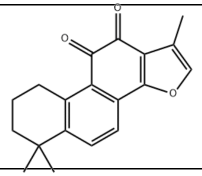	$C_{19}H_{18}O_3$	In vivo	In $ApoE^{-/-}$ mice	30 mg/kg/d and 10 mg/kg/d	Reducing in situ superoxide anion production, aortic expression of NF-κB and MMP-9.	Xu et al. [10]
				In vitro	RAW264.7 macrophages	10 μM, and 1 μM	Suppressing oxidized LDL-induced reactive oxygen species production, pro-inflammatory cytokine (IL-6, TNF-α, MCP-1) expression, and MMP-9 activity	Xu et al. [10]
				In vivo	In $ApoE^{-/-}$ mice	30 mg/kg/d and 60 mg/kg/d	Activating the estrogen receptor through the ERK signaling pathway	Liu et al. [11]
				In vivo	In rats	60 mg/kg/d	Reducing expression of MCP-1, TGF-β_1 and macrophage infiltration	Ren et al. [12]
				In vitro	In cardiac fibroblasts		Reducing MCP-1 and TGF-β_1 secretion	Ren et al. [12]
				In vivo	In $ApoE^{-/-}$ mice	60 mg/kg/d	Reduction C-reactive protein (CRP), ox-LDL, IL-1β, IL-6, IL-12, and TNF-α	Xuan et al. [13]
				In vivo	In rabbits	6.25, 15.00 and 37.50 mg/kg BW/day	Increasing SOD activity, decreasing MDA , CD40 and MMP-2 activity	Fang et al. [14]
				In vivo	In $ApoE^{-/-}$ mice	30 mg/kg/d	Reducing IL-1β, IL-6, MCP-1, and TNF-α	Wang et al. [15]

Table 1 (Continued) Anti-inflammation effects of Plant-Derived Natural Products and the underlying molecular mechanism.

TCM	Constituent	Chemical formula	Molecular formula	Study type	Subjects	Dose	Potential mechanism	References
				In vitro	In VSMCs	40 or 80 μM	Decreasing protein levels of MMP-9 and MMP-2	Wang et al. [15]
				In vitro	In RAW264.7 cells	40 or 80 μM	Reducing IL-1β, IL-6, MCP-1, and TNF-α	Wang et al. [15]
				In vitro	In DCs	1 μg/mL	Reducing IL-12 and IL-1	Li et al.[16]
				In vivo	In $ApoE^{-/-}$ mice	20 mg/kg/day	Inhibiting NF-κB activation to down regulate pro-IL -1β and NLRP3 expression	Wen et al. [17]
				In vivo	In $ApoE^{-/-}$ mice	10 mg/kg	Activating the TGF-β/PI3K/Akt/eNOS pathway	Wang et al. [18]
				In vivo	In $ApoE^{-/-}$ mice	10 mg/kg	Involving in the signaling pathways of Ras, Rap1, MAPK, cAMP, T cell receptor	Chen et al.[19]
				In vivo	In $ApoE^{-/-}$ mice	10 mg/kg	Via miR-375/KLF4 pathway	Chen et al. [20]
				In vitro	In Raw264.7 cells	10 μg/μl	Via miR-375/KLF4 pathway	Chen et al. [20]
				In vivo	In $LDLR^{-/-}$mice	7.5 mg/kg and 15 mg/kg	via down-regulation of MAPKs/NF-κB signaling pathway	Zhang et al. [21]
				In vitro	In RAW264.7 cells	20 μmol/L and 40 μmol/L	via down-regulation of MAPKs/NF-κB signaling pathway	Zhang et al. [21]
				In vitro	HUVECs	5μM	inhibiting NF-kapaB pathway via circ_0000231/miR-590-5p/TXNIP axis	Chen Z et al. [22]

Table 1 (Continued) Anti-inflammation effects of Plant-Derived Natural Products and the underlying molecular mechanism.

**TCM**	**Constituent**	**Chemical formula**	**Molecular formula**	**Study type**	**Subjects**	**Dose**	**Potential mechanism**	**References**
	DSS	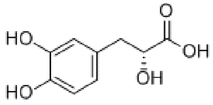	$C_9H_{10}O_5$	In vivo	In ApoE$^{-/-}$ mice	3 and 10 mg/kg/d	Inhibiting the toll-like receptor 4/nuclear factor kappa B pathway	Song et al. [23]
				In vitro	In BMMs	0.25, 0.5, and 1 μl/ml	Inhibiting the MAPK signaling pathway	Ye et al. [24]
	Rosmarinic acid	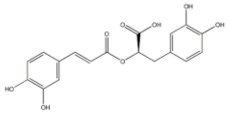	$C_{18}H_{16}O_8$	In vitro	In human endothelial cell line EA.hy926	1, 10, 50 or 100 μM	Down regulating the p38-FOXO1-TXNIP pathway	Nyandwi JB et al. [25]
	Sodium danshensu	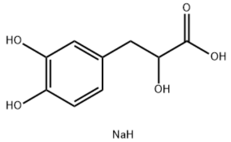	$C_9H_{11}NaO_5$	In vivo	In ApoE$^{-/-}$ mice	30 mg/kg or 60 mg/kg	via the miR-200a-3p/MEKK3/NF-κB Signaling Pathway	Zhang et al. [26]
				In vivo	In ApoE$^{-/-}$ mice	40 mg/kg/d and 80 mg/kg/d	targeting IKKβ mediated inflammation in macrophages	Zeng et al. [27]
				In vitro	In J774A.1 cells	12.5 μM、25 μM、50 μM、100 μM	targeting IKKβ mediated inflammation in macrophages	Zeng et al. [27]

Table 1 (Continued) Anti-inflammation effects of Plant-Derived Natural Products and the underlying molecular mechanism.

TCM	Constituent	Chemical formula	Molecular formula	Study type	Subjects	Dose	Potential mechanism	References
Ligusticum chuanxiong	TMP	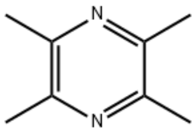	$C_8H_{12}N_2$	In vivo	In Sprague-Dawley rats	20 and 80 mg/kg	Restoring the total antioxidant capacity and superoxide dismutase 1 (SOD1) activity while decreasing the MDA generation	Jiang et al. [28]
				In vivo	In FPN1 Tek-Cre Mice	40 mg/kg	Modulating of hepcidin-ferroportin signaling	Sun et al. [29]
				In vivo	In rabbits	75 and 150 mg/kg	Decreasing the MCP-1 and ICAM-1 levels	Wang et al. [30]
				In vitro	In BMMs and HUVECs	250μM	Decreasing inflammation	Ye et al. [32]
				In vitro	In HUVECs	30, 60 and 120 μg/ml	Blocking ERK, p38 and nulear factor-κB signaling pathways	Li et al. [33]
	Ferulic acid	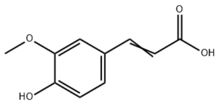	$C_{10}H_{10}O_4$	In vivo	In $ApoE^{-/-}$ mice	40 mg/kg/day	Via the AMPKα/SREBP1/ACC1 pathway	Gu et al. [34]
				In vitro	In VSMCs	200 and 400 ng/ml	Inhibiting VSMC Proliferation Through the NO/p21 Signaling pathway	Wu et al. [35]
Coptis chinensis	Berberine	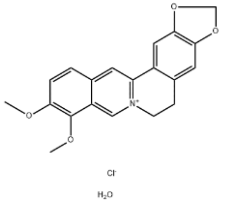	$C_{20}H_{18}NO_4$	In vivo	In $ApoE^{-/-}$ mice	100 and 50 mg/kg	Decreasing TNF-α, IL-1β, IL-6 and increasing IL-10 and adiponectin	Wu et al. [43]

Table 1 (Continued) Anti-inflammation effects of Plant-Derived Natural Products and the underlying molecular mechanism.

TCM	Constituent	Chemical formula	Molecular formula	Study type	Subjects	Dose	Potential mechanism	References
				In vivo	In $ApoE^{-/-}$ mice	0.5 g/L	Lowering expression of proinflammatory cytokines and chemokines	Zhu et al. [45]
				In vivo	In $ApoE^{-/-}$ mice		Enhancing the interplay between KLF16 and PPARα	Man et al. [46]
				In vivo	In $ApoE^{-/-}$ mice	78, 117 and 156 mg/kg	Through PI3K/AKTmTOR signaling pathway	Song et al. [47]
				In vivo	In $ApoE^{-/-}$ mice	5 mg/kg/day	Reducing visfatin, lipid, IL-6 and TNF-αand inhibiting p38 MAPK and JNK signaling pathways	Wan et al. [48]
				In vitro	in HUVECs	50 µmol/l	Decreasing apoptosis, IL-6 and TNF-α and inhibiting p38 MAPK and JNK signaling pathways	Wan et al. [48]
				In vivo	In $ApoE^{-/-}$ mice	150 mg/kg/d	Decreasing IL-1β and TNF-α as well as mRNA levels of NF-κBp65, i-NOS, ICAM-1, IL-6 in the aorta	Feng et al. [49]
				In vivo	In $ApoE^{-/-}$ mice	100mg / kg	Through the RAGE-NF-κB pathway	Zhang et al. [50]
				In vitro	In HUVEC	20µg/mL 、40µg/mL and 80µg/mL	Through the RAGE-NF-κB pathway	Zhang et al. [50]
				In vivo	In $ApoE^{-/-}$ mice	156 mg/kg/day	Through RXRα/PPARγ/NEDD4 Pathway	Zheng et al. [53]

Table 1 (Continued) Anti-inflammation effects of Plant-Derived Natural Products and the underlying molecular mechanism.

TCM	Constituent	Chemical formula	Molecular formula	Study type	Subjects	Dose	Potential mechanism	References
				In vitro	In RAW264.7 and peritoneal macrophage-derived foam cells	100 μmol/L	Through RXRα/PPARγ/NEDD4 Pathway	Zheng et al. [53]
				In vitro	In J774A.1 macrophages	6.25, 12.5, 25 and 50 μM	Alleviating inflammatory factors by up-regulation of autophagy via AMPK/mTOR signaling pathway	Fan et al. [51]
				In vitro	In Human peripheral blood mononuclear cells	5,10, 25, 50,75μM	Attenuating NLRP3 Inflammasome Activation in Macrophages to Reduce the Secretion of IL-1β	Jiang et al. [52]
	Coptisine	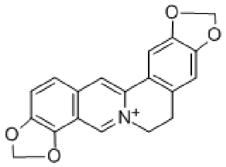	$C_{19}H_{14}NO_{4+}$	In vivo	In $ApoE^{-/-}$ mice	150 mg/kg/d	Decreasing TC, TG , LDL-C ,IL-6, IL-1β and TNF-α	Feng et al. [55]
				In vitro	In RAW 264.7 macrophages	30 μM	Inhibiting inflammation by blocking nuclear factor-kappa B, MAPK, and PI3K/Akt activation in macrophages	Wu et al. [56]

Table 1 (Continued) Anti-inflammation effects of Plant-Derived Natural Products and the underlying molecular mechanism.

TCM	Constituent	Chemical formula	Molecular formula	Study type	Subjects	Dose	Potential mechanism	References
Pseudo-ginseng	Notoginsenoside R1	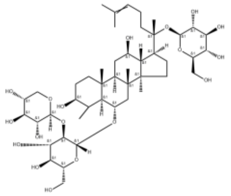	$C_{47}H_{80}O_{18}$	In vitro	In HUVECS	1, 10, and 30 μM	Alleviating apoptosis, inflammatory response, and oxidative stress in HUVECS through modulation of XIST/miR-221-3p/TRAF6 axis	Zhao et al. [58]
				In vivo	In ApoE$^{-/-}$ mice	25 mg/Kg	Reducing IL-2, IL-6, TNF-α and γ-IFN	Jia et al. [57]
				In vitro	In HUVECs	30 μM	Upregulating miR-221-3p expression to alleviate apoptosis, inflammation, and oxidative stress by inhibiting the TLR4/NF-κB pathway	Zhu et al. [59]
				In vivo	In ApoE$^{-/-}$ mice	10 mg/kg	Reducing the secretion of inflammatory factors, enhanced autophagy	Liu et al. [61]
				In vitro	In HAECs	Not mentioned	Regulating autophagy through the AMPK pathway	Liu et al. [61]
				In vivo	In Sprague-Dawley rats	25 mg/kg	Via Alleviating Inflammatory Response, Inhibiting Endothelial Dysfunction, and Regulating Gut Microbiota	Ma et al. [62]

Table 1 (Continued) Anti-inflammation effects of Plant-Derived Natural Products and the underlying molecular mechanism.

TCM	Constituent	Chemical formula	Molecular formula	Study type	Subjects	Dose	Potential mechanism	References
	Ginsenoside Rg1	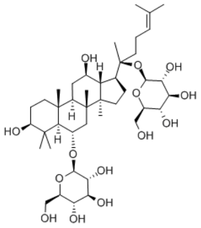	$C_{42}H_{72}O_{14}$	In vitro	In Raw264.7 macrophages	50 µM	Inhibiting apoptosis by increasing autophagy via the AMPK/mTOR signaling	Yang et al. [64]
	Quercetin	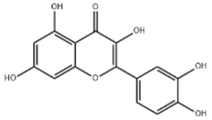	$C_{15}H_{10}O_{7}$	In vivo	In ApoE$^{-/-}$ mice	12.5 mg/(kg•d	Reducing PCSK9, TNF-α and IL-6	Li et al. [69]
				In vivo	In ApoE$^{-/-}$ mice	12.5 mg/kg/day	Reducing TNF-α, IL-1β, IL-18 and mTOR	Cao et al. [70]
				In vivo	In ApoE$^{-/-}$ mice	12.5 mg/kg/day	Regulating the expression of PCSK9, CD36, PPARγ, LXRα and ABCA1	Jia et al. [71]
				In vitro	In RAW264.7 cells	1, 5, and 10 µM	Reducing the expression levels of the M1 markers, such as IL-6, TNF-α, and IL-1β	Tsai et al. [72]
				In vivo	In ApoE$^{-/-}$ mice	100 mg $(kg{\cdot}bw)^{-1}$	Inhibiting Galectin-3-NLRP3 Signaling Pathway	Li et al. [73]
				In vivo	In Wistar rats	30 mg/kg/day	Modulating MPK/SIRT1/NF-κB signaling to inhibit inflammatory/oxidative stress responses	Zhang et al. [74]

Table 1 (Continued) Anti-inflammation effects of Plant-Derived Natural Products and the underlying molecular mechanism.

**TCM**	**Constituent**	**Chemical formula**	**Molecular formula**	**Study type**	**Subjects**	**Dose**	**Potential mechanism**	**References**
				In vivo	In Ldlr and Piezo1 endothelial-specific knockout mice	50 mg/kg and 100 mg/kg	Inhibiting inflammation of vascular endothelial cells via Piezo1 channels	Wang et al. [75]
				In vitro	In HUVECs	25 μM	Inhibiting inflammation of vascular endothelial cells via Piezo1 channels	Wang et al. [75]
				In vivo	In C57BL/6 mice	50 and 100 mg/kg	Suppressing inflammation and apoptosis via ROS-regulated PI3K/AKT signaling pathway	Lu et al. [76]
Radix Scutellariae	Baicalin	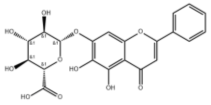	$C_{21}H_{18}O_{11}$	In vivo	In $ApoE^{-/-}$ mice	100 mg/kg	Increasing splenic Treg cells and the correlated cytokines (TGF-β1 and IL-10)	Liao et al. [77]
				In vivo	In $ApoE^{-/-}$ mice	50 and 100 mg/kg/d	Relieving oxidative stress and inflammatory responses via inactivating the NF-κB and p38 MAPK signaling pathways	Wu et al. [78]
				In vivo	In $ApoE^{-/-}$ mice	2, 50 and 100 mg/kg	Inhibiting NLRP3 inflammasome	Zhao et al. [80]
				In vitro	In HUVECs	0, 50, 100, and 150μM	Inhibiting PAR-1 expression and its downstream NF-κB activation	Zhang et al. [81]

Table 1 (Continued) Anti-inflammation effects of Plant-Derived Natural Products and the underlying molecular mechanism.

TCM	Constituent	Chemical formula	Molecular formula	Study type	Subjects	Dose	Potential mechanism	References
				In vitro	In VSMCs	20 μM	Upregulating miR-126-5p by targeting HMGB1	Chen et al. [82]
				In vivo	In $ApoE^{-/-}$ mice	100mg/kg/d	Increasing Wnt1 and inhibiting dickkopf-related protein-1 expression	Wang et al. [83]
	Baicalein	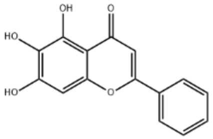	$C_{15}H_{10}O_5$	In vitro	In THP-1 macrophage-derived foam cells	0, 25, 50, 100, or 200 μM	Inhibiting lipid accumulation and inflammatory response by activating the PPARγ/LXRα pathway	Zhang et al. [87]
				In vitro	In RAW264.7, HUVEC, and MOVAS cells	1 μM	Targeting inflammation-associated AMPK//Mfn-2/MAPKs signaling pathways	Zhang et al. [88]
				In vitro	In HUVECs	2.5-20 μM	Protecting against oxidative stress and inflammation by modulation of AMPK-alpha	Tsai et al. [89]
	Wogonin	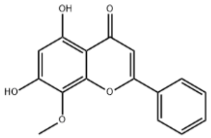	$C_{16}H_{12}O_5$	In vitro	In HUVECs	5 μM	Inhibiting MCP-1 and IL-8 mRNA and ROS formation.	Ku et al. [90]
				In vivo	In mice	1.1, 2.7, or 5.4 μg/mouse	Inhibiting reactive oxygen species (ROS) and NF-κB.	Ku et al. [90]

Table 1 (Continued) Anti-inflammation effects of Plant-Derived Natural Products and the underlying molecular mechanism.

TCM	Constituent	Chemical formula	Molecular formula	Study type	Subjects	Dose	Potential mechanism	References
				In vitro	In VSMCs	10, 25 and 50 μmol/L	Ameliorating apoptosis via interfering with DAG-PKC pathway	Liu et al. [91]
				In vivo	In LDLR$^{-/-}$ mice	10 mg day−1 kg−1body weight and 25 mg day−1 kg−1body weight	Via KLF11-Mediated Suppression of PPARα-YAP1-Driven Glycolysis and Enhancement of ABCA1/G1-Mediated Cholesterol Efflux	Ma et al. [92]
				In vitro	In RAW264.7 cells	--	Via KLF11-Mediated Suppression of PPARα-YAP1-Driven Glycolysis and Enhancement of ABCA1/G1-Mediated Cholesterol Efflux	Ma et al. [92]
Kudzu vine root	Puerarin	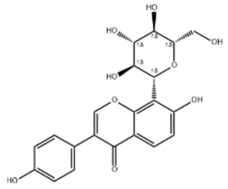	$C_{21}H_{20}O_9$	In vivo	In consecutive patients	400mg	Not mentioned	Yang et al. [93]
				In vivo	In rabbits	0.4g, 0.2g, 0.1 $g.kg^{-1}.d^{-1}$	Decreasing PCNA and PDGF-A expressions	Bao et al. [94]
				In vivo	In mice	50, 100, and 200 μM/kg/d)	Inhibiting the proliferation and inflammation through the miR-29b-3p/IGF1 pathway	Li et al. [95]

Table 1 (Continued) Anti-inflammation effects of Plant-Derived Natural Products and the underlying molecular mechanism.

TCM	Constituent	Chemical formula	Molecular formula	Study type	Subjects	Dose	Potential mechanism	References
				In vitro	In hVSMC	50, 100, and 200 μM	Inhibiting the proliferation and inflammation through the miR-29b-3p/IGF1 pathway	Li et al. [95]
				In vivo	In $ApoE^{-/-}$ mice	300、600、1200 mg.kg−1/d−1	Inhibiting oxidative stress disorder by activating JNK pathway and alleviates atherosclerosis	Li et al. [96]
				In vivo	In mice	100 mg/kg /day	Activatiing ERK5/KLF2 signaling pathway	Deng et al. [97]
				In vitro	In HUVECs	10 μM and 50 μM	Activatiing ERK5/KLF2 signaling pathway	Deng et al. [97]
				In vivo	In rabbits	20 mg/kg/d	Inhibiting the inflammatory response via modulation of the NF-κB pathway	Ji et al. [99]

Table 1 (Continued) Anti-inflammation effects of Plant-Derived Natural Products and the underlying molecular mechanism.

TCM	Constituent	Chemical formula	Molecular formula	Study type	Subjects	Dose	Potential mechanism	References
				In vitro	In peripheral blood mononuclear cells	10, 20 and 50 μm	Inhibiting C-reactive protein expression via suppression of nuclear factor kappaB activation	Yang et al. [100]
Ginseng	Ginsenoside Rb1	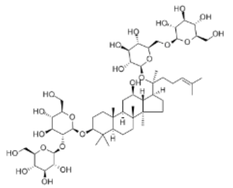	$C_{54}H_{92}O_{23}$	In vivo	In ApoE$^{-/-}$ mice	10 mg/kg	Inhibiting Apoptosis and Enhancing Autophagy	Zhou et al. [102]
				In vivo	In ApoE$^{-/-}$ mice	50 mg/kg/d	Anti-angiogenic and anti-inflammation effects exerted via the modulation of miR-33 and its target gene PEDF	Yang et al. [103]
				In vivo	In ApoE$^{-/-}$ mice	50 mg/kg/d	Improving Autophagy and Lipid Metabolism	Qiao et al. [86]
				In vitro	In macrophages	10, 20, 40, 80μm	Improving Autophagy and Lipid Metabolism	Qiao et al. [104]
				In vivo	In ApoE$^{-/-}$ mice	50 mg/kg/d	Increasing IL-4 and/or IL-13 and STAT6 phosphorylation	Zhang et al. [105]
				In vitro	In RAW264.7 cells	20μm	Promoting anti-inflammatory M2 macrophage polarization	Zhang et al. [105]
				In vitro	In HUVECs	20 μg/ml	Attenuating Inflammatory Injury via	Zhou et al.

							Inhibiting NF-κB, JNK and p38 Signaling Pathways	[106]

Table 1 (Continued) Anti-inflammation effects of Plant-Derived Natural Products and the underlying molecular mechanism.

**TCM**	**Constituent**	**Chemical formula**	**Molecular formula**	**Study type**	**Subjects**	**Dose**	**Potential mechanism**	**References**
				In vivo	In rabbits	5, 10, 20 mg/kg	Suppressing Inflammatory Response and Apoptosis via the GPER-mediated PI3K/Akt Pathway	Yang et al. [107]
				In vitro	In ECs	20, 40, 80 μM	Suppressing Inflammatory Response and Apoptosis via the GPER-mediated PI3K/Akt Pathway	Yang et al. [107]
	Ginsenoside Rh1	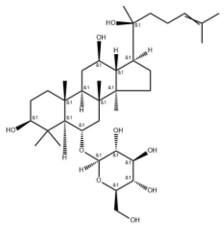	$C_{36}H_{62}O_9$	In vitro	In ECs	25 and 50 μM	Attenuating inflammatory injury through inhibiting TLR2/4-mediated STAT3, NF-κB, and ER stress signaling pathways	Jin et al. [108]
	Ginsenoside Rg2	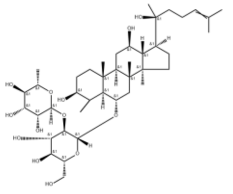	$C_{42}H_{72}O_{13}$	In vitro	In VSMCs	10 and 20 μM	Inhibiting the proliferation, migration, and phenotypic transformation induced by PDGF-BB	Xue et al. [109]
				In vitro	In HUVECs	10 and 20 μM	Decreasing inflammatory factors via blocking regulation of NF-κB and p-ERK signaling pathway	Xue et al. [109]

Table 1 (Continued) Anti-inflammation effects of Plant-Derived Natural Products and the underlying molecular mechanism.

TCM	Constituent	Chemical formula	Molecular formula	Study type	Subjects	Dose	Potential mechanism	References
				In vivo	In Sprague-Dawley rats	8 and 40 mg/kg/d	Reducing intimal proliferation after injury, regulating the inflammatory pathway to reduce inflammatory response	Xue et al. [109]
				In vitro	In HUVECs	1, 10, 20, 50 and 100 mol/l	Inhibiting Adhesion Molecule Expression	Cho et al. [110]
	Ginsenoside Rg3	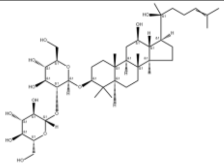	$C_{42}H_{72}O_{13}$	In vitro	In HUVECs	30 μM	Regulating PPARγ/FAK Signaling Pathway	Geng et al. [111]
				In vivo	In ApoE$^{-/-}$ mice	15, 30 mg/kg	Regulating PPARγ/FAK Signaling Pathway	Geng et al. [111]
				In vitro	In BMDMs	25 μM	Promoting macrophages to a profile of the M2 type through PPARγ-dependent mechanisms	Guo et al. [112]
				In vivo	In ApoE$^{-/-}$ mice	10 mg/kg/2d	Promoting macrophages to a profile of the M2 type through PPARγ-dependent mechanisms	Guo et al. [113]
Rhodiola	Salidroside	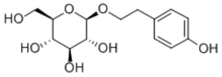	$C_{14}H_{20}O_7$	In vivo	In ApoE$^{-/-}$ mice	100 mg/kg/d	Via the cAMP/PKA/RhoA Signaling Pathway	Li et al. [116]

Table 1 (Continued) Anti-inflammation effects of Plant-Derived Natural Products and the underlying molecular mechanism.

TCM	Constituent	Chemical formula	Molecular formula	Study type	Subjects	Dose	Potential mechanism	References
				In vitro	In HUVECs	10 or 100 μM	Via the cAMP/PKA/RhoA Signaling Pathway	Li et al. [116]
				In vitro	In HUVECs	1μm	Inhibiting LDL transcytosis through enhancing the autophagic degradation of active Src and caveolin-1	Bai et al. [117]
				In vivo	In $ApoE^{-/-}$ mice	50 mg/kg/day	Inhibiting LDL transcytosis through enhancing the autophagic degradation of active Src and caveolin-1	Bai et al. [117]
				In vitro	In HUVECs	20, 50 and 100 μM	Enhancing autophagy mediated by SIRT1-FoxO1 pathway	Zhu et al. [118]
				In vitro	In macrophages	0.1, 1, 10 μM	Suppressing foam cell formation and apoptosis via the MAPK and AKT signaling pathways	Ni et al. [119]
				In vivo	In $ApoE^{-/-}$ mice	25 and 50 mg/kg/day	Activating a mitochondria-related AMPK/PI3K/Akt/eNOS pathway	Xing et al. [120]
				In vitro	In RAW264.7 cells	400 μM	Decreasing ROS levels, inhibiting NF-κB activation, regulating the expression of proinflammatory factors and mitochondrial homeostasis-associated proteins	Wang et al. [121]

Table 1 (Continued) Anti-inflammation effects of Plant-Derived Natural Products and the underlying molecular mechanism.

TCM	Constituent	Chemical formula	Molecular formula	Study type	Subjects	Dose	Potential mechanism	References
				In vivo	In mouse	50 mg/kg	Decreasing the expression of inflammatory cytokines and increasing the expression of SIRT3	Xing et al. [122]
				In vivo	In ApoE$^{-/-}$ mice	25 and 50 mg/kg	Inhibiting NLRP3-related pyroptosis	Xing et al. [123]
				In vitro	In HUVECs	1 and 10 μM	Inhibiting NLRP3-related pyroptosis	Xing et al. [123]
				In vitro	In CMECs	100 μM	Blocking mitogen-activated protein kinase and NF-κB signaling activation	Li et al. [124]
Angelica sinensis	Ligustilide	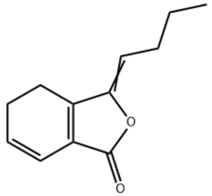	$C_{12}H_{14}O_2$	In vitro	In HUVECs	0.1–20 μM	Attenuating vascular inflammation and activating Nrf2/HO-1 induction and, NO synthesis	Choi et al.[114]
	Angelica sinensis polysaccharides			In vitro	In H9c2 cells	50 μg/ml	Activating ATF6 via AMPK-PGC1α pathway	Niu et al. [115]

Table 1 (Continued) Anti-inflammation effects of Plant-Derived Natural Products and the underlying molecular mechanism.

TCM	Constituent	Chemical formula	Molecular formula	Study type	Subjects	Dose	Potential mechanism	References
Radix Astragali	Astragaloside	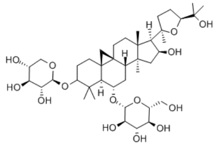	$C_{41}H_{68}O_{14}$	In vitro	In VSMCs	49 µg/mL	Regulating miR-17-5p and PCSK9/VLDLR signal pathway	Qin et al. [125]
				In vivo	In $ApoE^{-/-}$ mice	5 mg/kg/d	Regulating miR-17-5p and PCSK9/VLDLR signal pathway	Qin et al. [125]
				In vitro	In HUVECs	50 µM, 75 µM, or 100 µM	through targeting circ_0000231/miR-135a-5p/CLIC4 axis	Shao et al. [126]
				In vitro	In VSMCs	10, 20, or 40 µM	Inactivating the NF-κB pathway via regulating HDAC9	Chen D et al. [127]
				In vitro	In RAW264.7 macrophages	40 mg/kg/day	Promoting autophagy	Tian et al. [128]
				In vitro	In VSMCs	10, 20, and 50 µM	Reducing Oxidative Stress and Inflammation	Zhu et al. [129]
